# Pattern Specification and Immune Response Transcriptional Signatures of Pericardial and Subcutaneous Adipose Tissue

**DOI:** 10.1371/journal.pone.0026092

**Published:** 2011-10-11

**Authors:** Frank H. Lau, Rahul C. Deo, Gregory Mowrer, Joshua Caplin, Tim Ahfeldt, Adam Kaplan, Leon Ptaszek, Jennifer D. Walker, Bruce R. Rosengard, Chad A. Cowan

**Affiliations:** 1 Center for Regenerative Medicine and Cardiovascular Research Center, Massachusetts General Hospital, Boston, Massachusetts, United States of America; 2 Department of Stem Cell and Regenerative Biology, Harvard Stem Cell Institute, Harvard University, Cambridge, Massachusetts, United States of America; 3 Division of Cardiology, Department of Medicine, Massachusetts General Hospital, Boston, Massachusetts, United States of America; 4 Division of Cardiac Surgery, Department of Surgery, Massachusetts General Hospital, Boston, Massachusetts, United States of America; University of Tor Vergata, Italy

## Abstract

Cardiovascular disease (CVD) remains the leading cause of morbidity and mortality in the United States. Recent studies suggest that pericardial adipose tissue (PCAT) secretes inflammatory factors that contribute to the development of CVD. To better characterize the role of PCAT in the pathogenesis of disease, we performed a large-scale unbiased analysis of the transcriptional differences between PCAT and subcutaneous adipose tissue, analysing 53 microarrays across 19 individuals. As it was unknown whether PCAT-secreted factors are produced by adipocytes or cells in the supporting stromal fraction, we also sought to identify differentially expressed genes in isolated pericardial adipocytes vs. isolated subcutaneous adipocytes. Using microarray analysis, we found that: 1) pericardial adipose tissue and isolated pericardial adipocytes both overexpress atherosclerosis-promoting chemokines and 2) pericardial and subcutaneous fat depots, as well as isolated pericardial adipocytes and subcutaneous adipocytes, express specific patterns of homeobox genes. In contrast, a core set of lipid processing genes showed no significant overlap with differentially expressed transcripts. These depot-specific homeobox signatures and transcriptional profiles strongly suggest different functional roles for the pericardial and subcutaneous adipose depots. Further characterization of these inter-depot differences should be a research priority.

## Introduction

Cardiovascular disease (CVD) has long been the leading cause of death in the U.S. and the developed world [1]. While efforts to decrease the impact of the major cardiovascular risk factors have yielded modest success, there are indications that CVD mortality rates may rise again because of the epidemic of obesity and obesity-related comorbidities such as diabetes, hypercholesterolemia, and hypertension [2] [3] [4].

The relationship between adipose tissue and disease remains a very active area of study, and efforts have further intensified since the discovery that adipose tissue is an active, multifunctional endocrine organ [5] [6]. In part because of the strong correlation between the size of the visceral adipose tissue (VAT) depot and the incidence of CVD, type 2 diabetes mellitus, and metabolic syndrome, attention has focused on understanding the molecular and functional differences between VAT and subcutaneous adipose tissue (SQAT) [7] [8] [9]. Compared to SQAT, human VAT secretes more interleukin-6 (IL-6), expresses more peroxisome proliferator-activated receptor gamma (PPARγ), and produces less adiponectin [10] [11] [12]. With regards to CVD, IL-6 has been particularly interesting because of its close association to inflammation, obesity, and coronary heart disease [13]. It is also a strong, independent marker for increased mortality in the setting of CAD [14].

Given the depot-specific functions seen in VAT and SQAT, we hypothesized that pericardial adipose tissue (PCAT) may play a distinct physiologic role. Anatomically, PCAT lies between the visceral and parietal pericardium and is therefore distinct from epicardial adipose tissue (EAT) [15]. Using data from the Framingham Heart Study, radiographic studies positively correlated PCAT volume with the incidence of coronary artery calcification, as well as the elevation of several metabolic risk factors [16] [17]. Similar results were found using data from several other patient populations [18] [19] [20] [21]. These studies, while well-designed, provide neither transcriptional nor protein-level insight into the physiology of PCAT. To our knowledge, we are unaware of any studies that did so. In contrast, EAT has been shown to both overexpress and oversecrete inflammatory markers [22] [23] [24] [25].

Given the absence of non-radiographic data regarding the physiology of PCAT, we designed our study to identify transcriptional differences between PCAT and SQAT, and also between isolated pericardial adipocytes (pcAds) and isolated subcutaneous adipocytes (sqAds). Our study revealed that these depots express specific patterns of homeobox genes. We further found that PCAT and pcAds both overexpress atherosclerosis-promoting chemokines. These depot-specific homeobox signatures and transcriptional profiles strongly suggest different functional roles for the pericardial and subcutaneous adipose depots.

## Results

Between June 2009 and March 2010, 19 patients undergoing elective cardiac operations at Massachusetts General Hospital were enrolled in this study. From these patients, RNA samples were isolated from SQAT, PCAT, sqAds, and pcAds, of which 53 samples (11 SQAT, 11 PCAT, 15 sqAds, and 16 pcAds) passed quality standards for hybridization to Affymetrix U133A Plus 2.0 microarrays. Using nuclear and lipid stains, we determined that our isolated adipocytes contained 96.7% single-nuclei adipocytes (Fig. S1).

**Figure 1:**
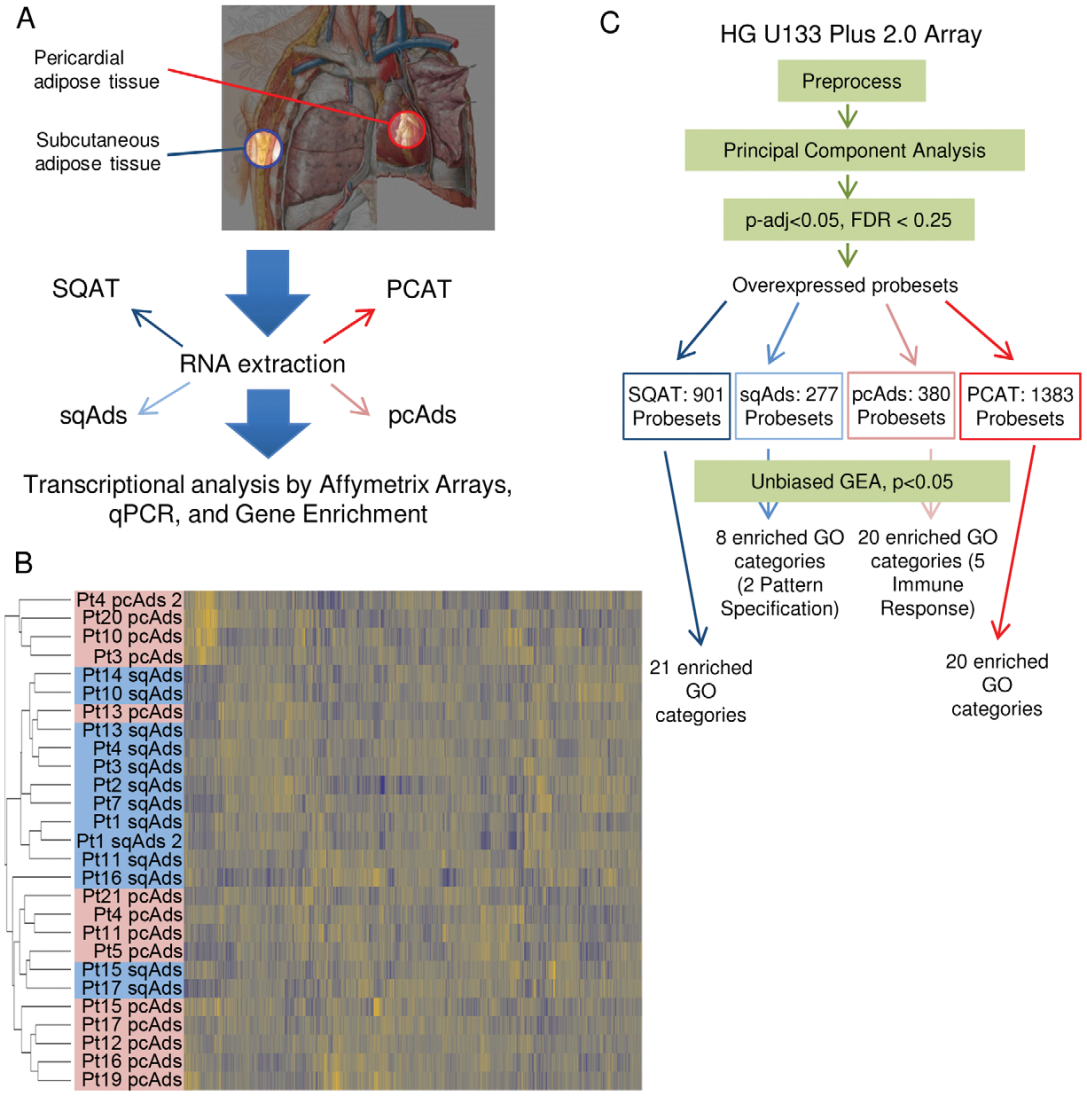
Experimental overview and clustering results. (A) Experimental design. (B) Clustering of isolated pericardial and subcutaneous adipocytes following removal of date-related batch effects by principal component analysis. (C) In our unbiased GEA, differentially expressed genes in each sample group (SQAT, sqAds, pcAds, and PCAT) were identified by preprocessing the data, performing principal component analysis, and setting thresholds of p-value<0.05 and FDR <0.25. Unique genes corresponding to candidate probesets were analyzed with FuncAssociate 2.0 (see Methods).

As our primary aim was the identification of depot-specific transcription patterns, we first performed unbiased hierarchical clustering of whole tissue and isolated adipocytes. This showed clustering of samples which were processed in one specific month, suggesting a strong batch effect (Fig. S2). To remove this effect, principal component analysis was performed. After correction for batch effects in processing (see Methods) clustering analysis revealed 3 distinct clusters. One cluster was composed solely of pcAds samples, a second cluster contained 10 samples of which 9 were pcAds, and a third cluster of 12 samples contained 9 pcAds samples (Fig. 1B).

We then used linear models to identify differentially expressed genes between populations PCAT and SQAT, and between pcAds and sqAds. Given the multiple hypothesis testing burden of microarray analyses, a nominal p-value of 0.05 almost certainly includes a large number of false positives. We thus restricted our analysis to those genes that were differentially at a false-discovery rate (FDR) of <0.25 (Fig. 1C, Methods). In PCAT vs. SQAT, this approach identified 2,284 differentially expressed probesets (Fig. 1C). In pcAds vs. sqAds, this analysis yielded 657 differentially expressed probesets.

To characterize the differentially expressed transcripts, we used unbiased gene enrichment analysis (GEA) to look for enriched Gene Ontology (GO) terms in the candidate probesets overexpressed in PCAT, SQAT pcAds and sqAds individually. Since multiple probesets map to the same genes, a list of unique candidate genes were generated from probeset lists. These candidate genes were ranked in order of greatest fold-change and analyzed for GO category enrichment as ordered lists using FuncAssociate 2.0. [26].

Our GEA revealed that the four tissue types were each enriched for multiple gene ontology categories (Table 1 and Table S1). Because we analyzed both whole tissue and isolated adipocytes, we were also able to infer whether the differentially expressed genes arise from predominantly either the adipocytes themselves, or from the more heterogeneous adipose tissue mixtures.

**Table 1:** Enriched Gene Ontology (GO) Categories in sqAds and pcAds identified by GEA.

# of Genes	P-adjusted	GO ID	GO Category
***Upregulated in sqAds***			
6	0.004	GO:0009952	anterior/posterior pattern formation
6	0.026	GO:0003002	regionalization
6	0.03	GO:0051262	protein tetramerization
8	0.047	GO:0051260	protein homooligomerization
10	0.006	GO:0043565	sequence-specific DNA binding
12	0.003	GO:0003700	transcription factor activity
19	0.02	GO:0009653	anatomical structure morphogenesis
48	0.002	GO:0032502	developmental process
***Upregulated in pcAds***			
27	0	GO:0048583	regulation of response to stimulus
38	0	GO:0042127	regulation of cell proliferation
39	0	GO:0002376	immune system process
62	0	GO:0048522	positive regulation of cellular process
67	0	GO:0048518	positive regulation of biological process
15	0.001	GO:0050865	regulation of cell activation
24	0.001	GO:0008284	positive regulation of cell proliferation
26	0.001	GO:0006955	immune response
14	0.005	GO:0006935	chemotaxis
14	0.005	GO:0042330	taxis
24	0.01	GO:0009605	response to external stimulus
27	0.013	GO:0005576	extracellular region
32	0.014	GO:0035466	regulation of signaling pathway
5	0.02	GO:0042692	muscle cell differentiation
11	0.023	GO:0009897	external side of plasma membrane
26	0.026	GO:0051239	regulation of multicellular organismal process
44	0.038	GO:0042221	response to chemical stimulus
11	0.04	GO:0046649	lymphocyte activation
13	0.042	GO:0001775	cell activation
21	0.044	GO:0002682	regulation of immune system process

In sqAds, our GEA revealed a striking enrichment of pattern specification genes (Table 1). Because the current GO classifications do not include all known homeobox genes, we manually referenced our differentially expressed genes against a comprehensive list of human homeobox genes [27]. Compared to pcAds, sqAds demonstrated increased expression of 12 homeobox genes, including PAX3, HOXA10, HOXA9, and HOXB7 (Table 1). All of the homeobox genes that were relatively higher in sqAds were also increased in SQAT vs. PCAT (Table S2). For example, PAX3 was 3.3-fold (p = 0.00002) increased in sqAds vs. pcAds and 3.8-fold (p = 0.0004) increased in SQAT vs. PCAT.

When we looked at homeobox expression in the pericardial depot, we found significantly increased expression of two homeobox genes, HOXA2 (2.53-fold, p = 0.00012) and SATB1 (1.84-fold, p = 0.015), in both pcAds and PCAT (Table 1 and Table S2). In aggregate, the observed transcriptional patterns of these 14 homeobox genes constitute depot-specific signatures.

**Figure 2:**
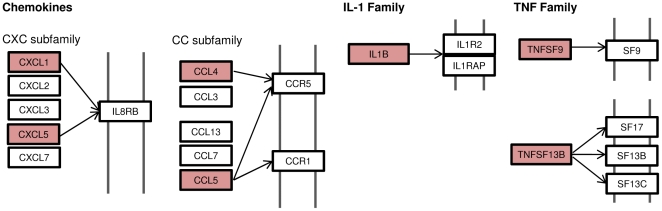
Chemokines overexpressed in pcAds. Red boxes represent overexpressed molecules. Double-gray lines indicate cell surface membrane; boxed proteins overlying double-gray lines are receptors.

GEA also found that our pcAds were significantly enriched for immune response genes (p-adj<0.001, Table 1 and Table S3), with 26 immune response genes found among the top 2500 genes. A similar result was seen in PCAT (p-adj = 0.005, Table S1). These genes included 7 chemokines, such as chemokine (C-C motif) ligand 4 (CCL4) which was overexpressed 2.79-fold (p<0.0001) in pcAds and 2.9-fold (p = 0.0009) in PCAT. To better visualize the molecular relationships between these inflammatory genes, we mapped the overexpressed genes to KEGG pathways (Fig. 2), revealing that the chemokines upregulated in pcAds belong to the tumor necrosis factor (TNF), CXC and CC chemokines, and IL-1 families. Since expression levels were not significantly lower in pcAds compared to PCAT, it is possible that pericardial adipocytes are a primary site for the synthesis of inflammatory mediators. We thus sought to determine, using qPCR, the expression of CCL4 in isolated pcAds and isolated pericardial stromal vascular fraction (SVF). We found 12.5-fold (p = 0.005) higher expression of CCL4 in the pericardial SVF vs. the pcAds fraction. Altogether, these data demonstrate that pcAds express inflammatory mediators but not to the same degree as the SVF, which contains immune and inflammatory cells. In contrast to PCAT, our GEA revealed that SQAT was significantly enriched in many metabolism-related GO categories (Table S1), including oxidoreductase activity (p-adj = 0.002) and lipid metabolic processes (p-adj = 0.008). KEGG pathway-mapping did not identify any recognized, adipose-tissue specific pathways.

**Figure 3:**
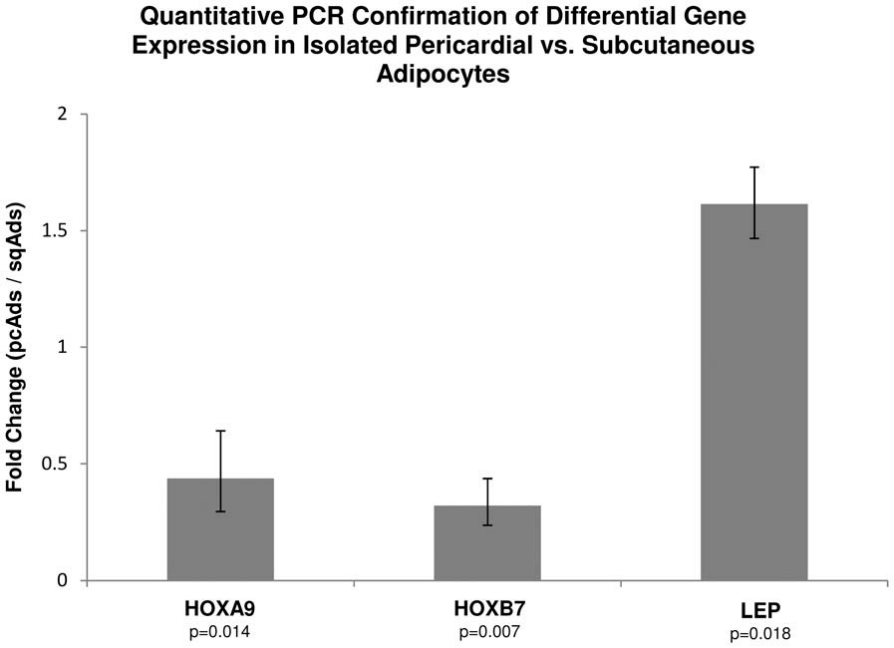
Quantitative PCR confirms differential expression of homeobox genes HOXA9 and HOXB7; and adipocyte-identity gene leptin (LEP) in patient-matched, isolated subcutaneous and pericardial adipocytes. Fold change patterns fit those seen in the microarray data. The error bars reflect the effect of a unit increase or decrease in standard deviation of the delta Ct value on the fold-change. P-values were calculated using 2-tailed heteroscedastic Student's t-tests.

To validate the differential gene expression patterns identified by our microarray analysis, we performed qPCR analysis on patient-matched sqAds and pcAds samples (Fig. 3). For this analysis, we selected homeobox genes HOXA9 and HOXB7; and the adipocyte identity gene leptin. For all three genes, the expression trend matched our microarray results.

Lastly, we confirmed that a core set of adipocyte identity and function genes was not differentially expressed in pcAds vs. sqAds (Table S4). The genes we selected included the well-studied adipocyte identity maintenance factors perilipin (PLIN1) and cell death-inducing DFFA-like effector c (CIDEC); the transcription factors Peroxisome proliferator-activated receptor gamma (PPARγ) and the CCAAT/enhancer binding proteins (CEBPA, CEBPB, CEBPD); the lipid catabolism enzymes lipoprotein lipase (LPL) and hormone-sensitive lipase (LIPE); the adipocyte anabolic enzymes fatty acid binding protein 4 (FABP4) and diacylglycerol O-acyltransferase 1 (DGAT1); and the adipokines leptin (LEP), adipsin (CFD), and adiponectin (ADIPOQ). We also considered the brown fat identity gene uncoupling protein 1 (UCP1) and found ≤0.50 fold change between the sample classes.

## Discussion

Adipose tissue is an active, multifunctional organ whose different depots in the human body most likely serve different functions. For example, a strong correlation exists between excess omental adipose tissue and the development of cardiovascular disease [7] [8] [9]. While early evidence suggested that pericardial adipose tissue functions in pericardial inflammation, these studies were purely radiographic in nature [16] [17] [18] [19] [20] [21]. There is evidence, however, that PCAT can directly affect cardiac function as there is a several studies have found an association between PCAT volume and atrial fibrillation [28] [29]. PCAT may also serve an endocrine role, as increased PCAT volume is positively associated with liver fat and insulin sensitivity after diet-induced weight loss [30].

Our study is the first transcriptional microarray analysis of gene expression differences between PCAT and SQAT and, importantly, between pcAds and sqAds. We found that: 1) pericardial and subcutaneous fat depots, as well as isolated pericardial and subcutaneous adipocytes, express unique patterns of homeobox genes, and 2) pericardial adipose tissue and isolated pericardial adipocytes both overexpress chemokines that promote atherosclerosis. In contrast, subcutaneous adipose tissue is enriched for metabolism-related genes.

The finding of a specific pericardial-specific homeobox signature fits with recent reports of depot-dependent homeobox signatures in humans. Gesta et al. compared human SQAT to visceral adipose tissue (VAT) and found that SQAT overexpressed SHOX2, HOXC9, EN1, and GPC4, while VAT overexpressed factors including HOXA5, TBX15, and HOXC8 [31]. Our data confirm that SHOX2 and HOXC9 are upregulated in SQAT (Table 2). Vohl et al. reported that HOXA10 and HOXC6 were upregulated in human SQAT vs. VAT; [32] this pattern is also confirmed by our data (Table 1).

**Table 2:** Upregulated Homeobox Genes in isolated adipocytes from the pericardial and subcutaneous depots.

*Overexpressed in pericardial adipocytes*			
Gene	p-value	q-value	Fold Change
HOXA2	0.00012	0.0471	2.53
SATB1	0.01486	0.2493	1.84

*Overexpressed in subcutaneous adipocytes*			
Gene	p-value	q-value	Fold Change
PAX3	0.00002	0.0304	3.30
IRX5	0.00002	0.0304	2.97
EMX2	0.00003	0.0337	2.65
HOXC9	0.00007	0.0405	2.56
HOXA10	0.00004	0.0337	2.50
IRX3	0.00031	0.0747	2.49
IRX1	0.00069	0.0967	2.48
HOXB7	0.00013	0.0475	2.45
HOXA9	0.00011	0.0471	2.39
HOXC6	0.00109	0.1171	2.30
SHOX2	0.02595	0.2920	1.97
HOXC10	0.01495	0.2493	1.79

The functional importance of these signatures needs further study. One hypothesis is that these homeobox signatures reflect distinct cell lineages [33] [34]. If so, such lineage differences, if extensive, may imply that excess adiposity of different fat depots (e.g. excess omental fat vs. excess pericardial fat) would not respond equally well to single therapies. Rather than simply being markers, homeobox genes could also function in adipose tissue function. Dankel et al. reported that profound fat loss in humans was correlated with strong upregulation in SQAT of HOXA9, HOXC6, and several other homeobox genes [35]. These genes could represent novel targets for modifying adipogenesis and, by extension, obesity.

The heterogeneous nature of different adipose depots is highlighted by the functional differences in this study. Our data show that both pericardial adipose tissue and isolated pericardial adipocytes are immunologically active, overexpressing several chemokines (Fig. 2), many of which have been implicated in the development of atherosclerosis and coronary artery disease (CAD).

The CC subfamily chemokines overexpressed in our pcAds (CCL4, and CCL5) are found at high levels in atherosclerotic lesions [36] [37] [38] [39] [40]. CCL5 has been indirectly shown to be atherogenic: antagonism of its receptor with a methionine-retaining CCL5 isoform slows atherosclerosis *in vivo* [41]. Although it was not included in Fig. 2, CCL2 also trended towards overexpression in pcAds (1.5-fold overexpression, p-value = 0.017) but had an FDR of 0.26. When CCL2, also known as monocyte chemoattractant protein-1 (MCP-1), was deleted in transgenic mice, macrophages were not recruited into atherosclerotic plaques and the atherosclerotic lesions were 60–70% smaller [42]. A similar phenotype was observed in mice deficient for CCR2, the receptor for CCL2 [43].

The overexpressed CXC subfamily chemokines also have direct, pro-atherosclerotic effects *in vivo* [44]. CXCL1 was implicated in the transition of early fatty streaks to intermediate atherosclerotic lesions [45]. In porcine models it induced endothelial dysfunction by downregulating nitric oxide synthase protein levels [46]. CXCL5 appears to elevate serum levels of CXCL1 and may be an indirect factor in atherogenesis [47].

In the interleukin family, IL-1β (1.7-fold overexpressed in pcAds) is known to increase LDL binding to atherosclerotic lesions which then induces more IL-1 production [48] [49]. Conversely, atherosclerosis-prone mice that were deficient in IL-1β had significantly smaller atherosclerotic plaques [50]. Given this mechanistic data, the overexpression of these chemokines in pcAds strongly suggests a direct role for pericardial adipocytes in the development of coronary artery atherosclerosis.

Our study highlights a number of new areas for further research. It is unclear if these depot-specific homeobox gene signatures are residual from differentiation, or continue to drive expression of genes important for defining tissue identity. Compelling follow-up studies would include identification of downstream targets and pathways of these homeobox genes. More research is also needed to confirm local and perhaps systemic levels of pericardial adipocyte-secreted chemokines, and further on, to identify the pathways that they activate.

In conclusion, pericardial and subcutaneous fat depots express unique homeobox signatures and serve very different functional roles. As the first microarray study of the pericardial adipose depot, we show that pericardial adipocytes preferentially express pro-atherosclerotic factors and may therefore function in the development of cardiovascular disease. Future research should aim to further characterize these emerging, depot-specific functions.

## Materials and Methods

This Project has been reviewed and approved by the Brigham & Women's Hospital Institutional Review Board, Assurance # FWA00000484. During the review of this Project, the IRB specifically considered (i) the risks and anticipated benefits, if any, to subjects; (ii) the selection of subjects; (iii) the procedures for securing and documenting informed consent; (iv) the safety of subjects; and (v) the privacy of subjects and confidentiality of the data. Written consent was obtained from all participants.

Between June 2009 and April 2010, 21 patients who underwent elective coronary artery bypass grafting and/or cardiac valve replacement surgeries participated in the study. With the exception of prisoners and subjects with surrogate decision makers, there were no exclusion criteria or patient classes. No enrolled patients were excluded. Approximately 5 mL of pericardial adipose tissue (PCAT) and 3 mL of subcutaneous adipose tissue (SQAT) from the anterior chest wall were harvested from patients undergoing elective coronary artery bypass grafting and/or cardiac valve replacement surgery. Epicardial adipose tissue was not harvested. As adipose degrades quickly after surgical removal, all samples were retrieved immediately from the operating room after being harvested. The samples were sectioned and assigned to either whole tissue analysis or isolated adipocyte analysis. For PCAT and SQAT samples, RNA was immediately extracted via submersion in 1 ml of Trizol, mechanical lysis using an RNase free pestle (Kimble Chase Kontes), addition of 200 µl chloroform, and centrifugation (10,000 g, 15 minutes, 4C). The supernatant was extracted and purified with RNeasy Mini Columns (Qiagen). 1.2 µg RNA was synthesized into complementary DNA (cDNA) with the Superscript III First Strand Synthesis Kit (Invitrogen).

For adipocyte isolation, tissue samples were minced and digested in Liberase Blendzyme (0.625 g dissolved in 50 mL PBS) for one hour at 37C, in a rotating incubator. Following digestion, Blendzyme was inactivated via the addition of KRB solution (bovine serum albumin, Hank's balanced salt solution, PBS, and gentamicin). The samples were filtered through a 200 micron steel mesh filter and centrifuged at 1000 rpm for 5 minutes. Single adipocytes were collected from the uppermost fraction and immediately frozen in liquid nitrogen. RNA was then extracted from the sqAds and pcAds samples in the same fashion as SQAT/PCAT samples. To quantify the purity of the isolated adipocytes, 150 uL of freshly isolated adipocytes was mixed with 150 uL of PBS containing Hoescht 33258 nuclear stain (Invitrogen, 10 mg/mL, diluted 1∶5000) and Bodipy stain (Invitrogen, 5 mM diluted 1∶20,000). 50 uL of this mix was mounted directly onto slides (Fischer) using Aqua Poly/Mount (Polysciences). Cells and nuclei were counted using a Nikon Eclipse Ti microscope, and representative images were captured using NIS-Elements software package (Nikon, version 3.10).

All RNA samples were analyzed for quality using an Agilent 2100 Bioanalyzer. Suitable samples were hybridized to Affymetrix Human Genome U133 Plus 2.0 Arrays. The arrays were processed by the Stowers Institute for Medical Research Bioinformatics Core Facility. All data is MIAME compliant and the raw data has been deposited in the Gene Expression Omnibus database (accession number GSE26339).

Raw expression values were analyzed with GenePattern 2.0 [51]. Data were normalized using Robust Multiarray Averaging (RMA) with median scaling, quintile normalization, and background correction. The resulting datasets were preprocessed to remove probesets whose minimum fold change (maximum gene expression value divided by the minimum value) was <2, or whose difference between maximum and minimum values was less than 100. The preprocessed data was clustered in an unbiased hierarchical fashion, with Pearson correlation and pairwise complete-linkage.

RNA samples were collected, extracted, amplified and hybridized in several separate batches over the period of 10 months. To explore the influence of batch effects, principal component analysis was performed using the *prcomp* function in R (2.9.1). The first principal component, explaining 38% of the variance, corresponded to the date of sample processing, with samples processed in June 2009 grouped separately from all other samples. The contribution of this component was removed from normalized intensities prior to further analysis.

Unbiased GEA was performed using FuncAssociate 2.0 (http://llama.mshri.on.ca/funcassociate/), which uses a Fisher's exact test to assess enrichment and a resampling approach to correct for multiple hypotheses. For each of the sample populations, a false discovery rate (FDR) of 0.25 was set as the threshold. We also performed sensitivity analysis to ensure that FDR thresholds between 0.2 and 0.3 did not change the identify of enriched gene sets. The differentially expressed probesets were uploaded into FuncAssociate 2.0 as ordered lists. Analysis was performed using the hgnc_symbol namespace, with 1000 permutations for p-value estimation and a p-value cutoff of 0.05.

Pathways involving upregulated genes in significantly enriched GO categories were identified using DAVID and KEGG [52] [53] [54].

For qPCR, expression levels of HOXA9 and HOXB7 were normalized to the housekeeping gene beta-2-microglobulin (B2M) and measured via Taqman Assay (Applied Biosciences). Both the pcAds and sqAds samples were patient-matched (Patient 15). Leptin expression levels in matched samples (from Patient 5) were normalized to hypoxanthine-guanine phosphoribosyltransferase (HPRT) and measured via Quantifast SYBR Green PCR Kit (Qiagen). To minimize the potential impact of any genomic DNA contamination, all primers were designed and verified to span multiple exons. Three technical replicates were performed for each sample. Error bars were computed by adding and subtracting 1 unit standard deviation of the delta Ct values from calibrated delta Ct values. P-values were calculated in Microsoft Excel 2007 using 2-tailed heteroscedastic Student's t-tests.

## Supporting Information

Figure S1 Purity of isolated adipocytes. (A) Results of nuclear and cell counts in isolated adipocyte fraction; the fraction is 96.7% pure. (B) Representative image of isolated sqAds demonstrating 1∶1 association of nuclei with adipocytes.
(TIF)

Figure S2 Unbiased hierarchical clustering of isolated pericardial adipocytes (pcAds) and isolated subcutaneous adipocytes (sqAds). Sections of the dendrogram in red indicate samples clustering of pcAds and sqAds samples processed in June 2009.
(TIF)

Table S1 Enriched Gene Ontology categories in SQAT and PCAT.
(DOCX)

Table S2 Upregulated Homeobox Genes in SQAT and PCAT.
(DOCX)

Table S3 Upregulated Immune Response genes in pericardial adipocytes.
(DOCX)

Table S4 Expression of core adipocyte function genes in isolated pericardial adipocytes (pcAds) vs. isolated subcutaneous adipocytes (sqAds).
(DOCX)

## References

[1] Ford ES, Capewell S (2007). Coronary Heart Disease Mortality Among Young Adults in the U.S. From 1980 Through 2002: Concealed Leveling of Mortality Rates.. Journal of the American College of Cardiology.

[2] Hardoon SL, Whincup PH, Lennon LT, Wannamethee SG, Capewell S (2008). How Much of the Recent Decline in the Incidence of Myocardial Infarction in British Men Can Be Explained by Changes in Cardiovascular Risk Factors?: Evidence From a Prospective Population-Based Study.. Circulation.

[3] FASTSTATS - Leading Causes of Death http://www.cdc.gov/nchs/fastats/lcod.htm.

[4] Berrington de Gonzalez A, Hartge P, Cerhan JR, Flint AJ, Hannan L (2010). Body-Mass Index and Mortality among 1.46 Million White Adults.. N Engl J Med.

[5] Zhang Y, Proenca R, Maffei M, Barone M, Leopold L (1994). Positional cloning of the mouse obese gene and its human homologue.. Nature.

[6] Flier JS (2004). Obesity wars: molecular progress confronts an expanding epidemic.. Cell.

[7] Després J-P, Lemieux I (2006). Abdominal obesity and metabolic syndrome.. Nature.

[8] Despres J, Moorjani S, Lupien P, Tremblay A, Nadeau A (1990). Regional distribution of body fat, plasma lipoproteins, and cardiovascular disease.. Arterioscler Thromb Vasc Biol.

[9] Trayhurn P, Wood IS (2004). Adipokines: Inflammation and the Pleiotropic Role of White Adipose Tissue.. British Journal of Nutrition.

[10] Fried SK, Bunkin DA, Greenberg AS (1998). Omental and subcutaneous adipose tissues of obese subjects release interleukin-6: depot difference and regulation by glucocorticoid.. J Clin Endocrinol Metab.

[11] Lefebvre AM, Laville M, Vega N, Riou JP, van Gaal L (1998). Depot-specific differences in adipose tissue gene expression in lean and obese subjects.. Diabetes.

[12] Fisher FM, McTernan PG, Valsamakis G, Chetty R, Harte AL (2002). Differences in adiponectin protein expression: effect of fat depots and type 2 diabetic status.. Horm Metab Res.

[13] Yudkin JS, Kumari M, Humphries SE, Mohamed-Ali V (2000). Inflammation, obesity, stress and coronary heart disease: is interleukin-6 the link?. Atherosclerosis.

[14] Lindmark E, Diderholm E, Wallentin L, Siegbahn A (2001). Relationship Between Interleukin 6 and Mortality in Patients With Unstable Coronary Artery Disease: Effects of an Early Invasive or Noninvasive Strategy.. JAMA.

[15] Iacobellis G (2009). Epicardial and Pericardial Fat: Close, but Very Different.. Obesity.

[16] Rosito GA, Massaro JM, Hoffmann U, Ruberg FL, Mahabadi AA (2008). Pericardial Fat, Visceral Abdominal Fat, Cardiovascular Disease Risk Factors, and Vascular Calcification in a Community-Based Sample: The Framingham Heart Study.. Circulation.

[17] Tadros TM, Massaro JM, Rosito GA, Hoffmann U, Vasan RS (2010). Pericardial Fat Volume Correlates With Inflammatory Markers: The Framingham Heart Study.. Obesity.

[18] Ding J, Kritchevsky SB, Harris TB, Burke GL, Detrano RC (2008). The Association of Pericardial Fat With Calcified Coronary Plaque.. Obesity.

[19] Kim TH, Yu SH, Choi SH, Yoon JW, Kang SM (2010). Pericardial Fat Amount Is an Independent Risk Factor of Coronary Artery Stenosis Assessed by Multidetector-Row Computed Tomography: The Korean Atherosclerosis Study 2.. Obesity.

[20] Liu J, Fox CS, Hickson D, Sarpong D, Ekunwe L (2010). Pericardial adipose tissue, atherosclerosis, and cardiovascular disease risk factors: the Jackson heart study.. Diabetes Care.

[21] Greif M, Becker A, von Ziegler F, Lebherz C, Lehrke M (2009). Pericardial adipose tissue determined by dual source CT is a risk factor for coronary atherosclerosis.. Arterioscler Thromb Vasc Biol.

[22] Mazurek T, Zhang L, Zalewski A, Mannion JD, Diehl JT (2003). Human epicardial adipose tissue is a source of inflammatory mediators.. Circulation.

[23] Dutour A, Achard V, Sell H, Naour N, Collart F (2010). Secretory type II phospholipase A2 is produced and secreted by epicardial adipose tissue and overexpressed in patients with coronary artery disease.. J Clin Endocrinol Metab.

[24] Baker AR, Silva NFda, Quinn DW, Harte AL, Pagano D (2006). Human epicardial adipose tissue expresses a pathogenic profile of adipocytokines in patients with cardiovascular disease.. Cardiovasc Diabetol.

[25] Cheng K-H, Chu C-S, Lee K-T, Lin T-H, Hsieh C-C (2008). Adipocytokines and proinflammatory mediators from abdominal and epicardial adipose tissue in patients with coronary artery disease.. Int J Obes (Lond).

[26] Berriz GF, Beaver JE, Cenik C, Tasan M, Roth FP (2009). Next generation software for functional trend analysis.. Bioinformatics.

[27] Holland PW, Booth HAF, Bruford EA Classification and nomenclature of all human homeobox genes.. BMC Biol.

[28] Thanassoulis G, Massaro JM, O'Donnell CJ, Hoffmann U, Levy D (2010). Pericardial fat is associated with prevalent atrial fibrillation: the Framingham Heart Study.. Circ Arrhythm Electrophysiol.

[29] Babcock MJ, Soliman EZ, Ding J, A Kronmal R, Goff DC (2011). Pericardial fat and atrial conduction abnormalities in the Multiethnic Study of Atherosclerosis (MESA).. Obesity (Silver Spring).

[30] Bosy-Westphal A, Kossel E, Goele K, Blocker T, Lagerpusch M (2010). Association of Pericardial Fat With Liver Fat and Insulin Sensitivity After Diet-Induced Weight Loss in Overweight Women.. Obesity.

[31] Gesta S, Blüher M, Yamamoto Y, Norris AW, Berndt J (2006). Evidence for a role of developmental genes in the origin of obesity and body fat distribution.. Proc Natl Acad Sci USA.

[32] Vohl M-C, Sladek R, Robitaille J, Gurd S, Marceau P (2004). A survey of genes differentially expressed in subcutaneous and visceral adipose tissue in men.. Obes Res.

[33] Chang HY (2009). Anatomic demarcation of cells: genes to patterns.. Science.

[34] Rinn JL, Wang JK, Liu H, Montgomery K, van de Rijn M (2008). A systems biology approach to anatomic diversity of skin.. J Invest Dermatol.

[35] Dankel SN, Fadnes DJ, Stavrum A-K, Stansberg C, Holdhus R (2010). Switch from stress response to homeobox transcription factors in adipose tissue after profound fat loss.. PLoS ONE.

[36] Nelken NA, Coughlin SR, Gordon D, Wilcox JN (1991). Monocyte chemoattractant protein-1 in human atheromatous plaques.. J Clin Invest.

[37] Ylä-Herttuala S, Lipton BA, Rosenfeld ME, Särkioja T, Yoshimura T (1991). Expression of monocyte chemoattractant protein 1 in macrophage-rich areas of human and rabbit atherosclerotic lesions.. Proc Natl Acad Sci USA.

[38] Yu X, Dluz S, Graves DT, Zhang L, Antoniades HN (1992). Elevated expression of monocyte chemoattractant protein 1 by vascular smooth muscle cells in hypercholesterolemic primates.. Proc Natl Acad Sci USA.

[39] Schecter AD, Calderon TM, Berman AB, McManus CM, Fallon JT (2000). Human vascular smooth muscle cells possess functional CCR5.. J Biol Chem.

[40] von Hundelshausen P, Weber KS, Huo Y, Proudfoot AE, Nelson PJ (2001). RANTES deposition by platelets triggers monocyte arrest on inflamed and atherosclerotic endothelium.. Circulation.

[41] Veillard NR, Kwak B, Pelli G, Mulhaupt F, James RW (2004). Antagonism of RANTES Receptors Reduces Atherosclerotic Plaque Formation in Mice.. Circ Res.

[42] Gosling J, Slaymaker S, Gu L, Tseng S, Zlot CH (1999). MCP-1 deficiency reduces susceptibility to atherosclerosis in mice that overexpress human apolipoprotein B.. J Clin Invest.

[43] Boring L, Gosling J, Cleary M, Charo IF (1998). Decreased lesion formation in CCR2−/− mice reveals a role for chemokines in the initiation of atherosclerosis.. Nature.

[44] Breland UM, Halvorsen B, Hol J, Øie E, Paulsson-Berne G (2008). A potential role of the CXC chemokine GROalpha in atherosclerosis and plaque destabilization: downregulatory effects of statins.. Arterioscler Thromb Vasc Biol.

[45] Boisvert WA, Rose DM, Johnson KA, Fuentes ME, Lira SA (2006). Up-regulated expression of the CXCR2 ligand KC/GRO-alpha in atherosclerotic lesions plays a central role in macrophage accumulation and lesion progression.. Am J Pathol.

[46] Bechara C, Wang X, Chai H, Lin PH, Yao Q (2007). Growth-related oncogene-alpha induces endothelial dysfunction through oxidative stress and downregulation of eNOS in porcine coronary arteries.. Am J Physiol Heart Circ Physiol.

[47] Mei J, Liu Y, Dai N, Favara M, Greene T (2010). CXCL5 regulates chemokine scavenging and pulmonary host defense to bacterial infection.. Immunity.

[48] Stopeck AT, Nicholson AC, Mancini FP, Hajjar DP (1993). Cytokine regulation of low density lipoprotein receptor gene transcription in HepG2 cells.. J Biol Chem.

[49] Ross R (1999). Atherosclerosis–an inflammatory disease.. N Engl J Med.

[50] Kirii H, Niwa T, Yamada Y, Wada H, Saito K (2003). Lack of Interleukin-1{beta} Decreases the Severity of Atherosclerosis in ApoE-Deficient Mice.. Arterioscler Thromb Vasc Biol.

[51] Reich M, Liefeld T, Gould J, Lerner J, Tamayo P (2006). GenePattern 2.0.. Nat Genet.

[52] Dennis G, Sherman BT, Hosack DA, Yang J, Gao W (2003). DAVID: Database for Annotation, Visualization, and Integrated Discovery.. Genome Biol.

[53] Kanehisa M, Goto S, Furumichi M, Tanabe M, Hirakawa M (2010). KEGG for representation and analysis of molecular networks involving diseases and drugs.. Nucleic Acids Res.

[54] Kanehisa M, Goto S (2000). KEGG: kyoto encyclopedia of genes and genomes.. Nucleic Acids Res.

