# Supplementary figures and images for: Pattern Specification and Immune Response Transcriptional Signatures of Pericardial and Subcutaneous Adipose Tissue
Source: PLoS One. 2011 Oct 11;6(10):e26092. doi: 10.1371/journal.pone.0026092 (PMC3191160; doi:10.1371/journal.pone.0026092)

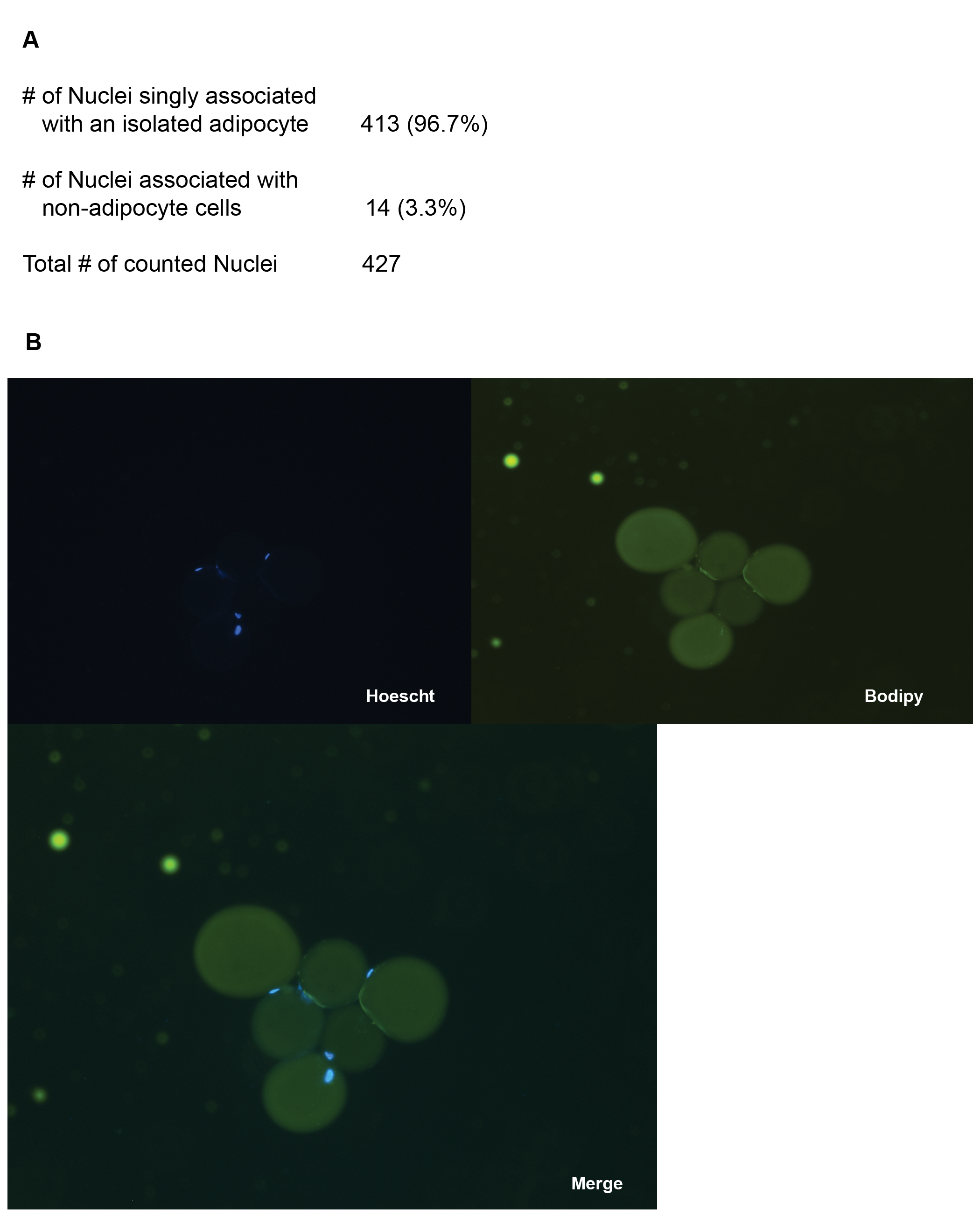

Supplement: Figure S1 — Purity of isolated adipocytes. (A) Results of nuclear and cell counts in isolated adipocyte fraction; the fraction is 96.7% pure. (B) Representative image of isolated sqAds demonstrating 1∶1 association of nuclei with adipocytes. (TIF) [file pone.0026092.s001.tif]

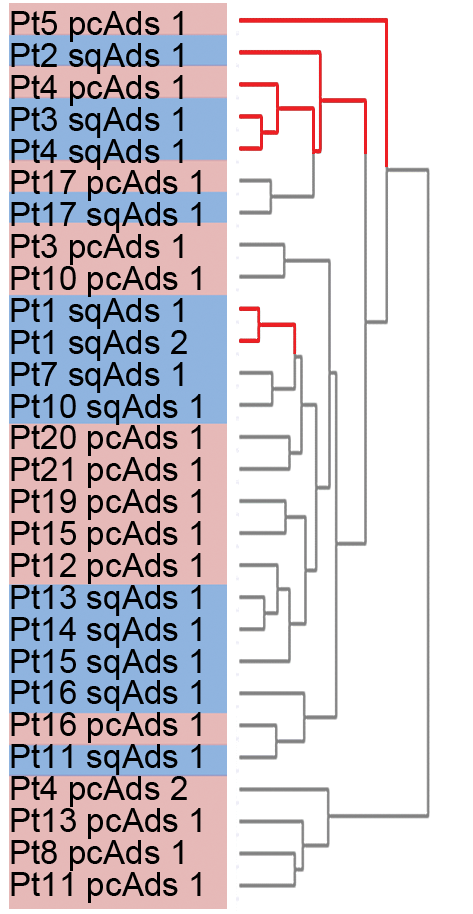

Supplement: Figure S2 — Unbiased hierarchical clustering of isolated pericardial adipocytes (pcAds) and isolated subcutaneous adipocytes (sqAds). Sections of the dendrogram in red indicate samples clustering of pcAds and sqAds samples processed in June 2009. (TIF) [file pone.0026092.s002.tif]
