# Supplementary material for: Pattern Specification and Immune Response Transcriptional Signatures of Pericardial and Subcutaneous Adipose Tissue
Source: PLoS One. 2011 Oct 11;6(10):e26092. doi: 10.1371/journal.pone.0026092 (PMC3191160; doi:10.1371/journal.pone.0026092)
Supplement: Table S1 — Enriched Gene Ontology categories in SQAT and PCAT. (DOCX) [file pone.0026092.s003.docx]

| # of Genes | P-adjusted | GO ID | GO Category |
| --- | --- | --- | --- |
| ***Upregulated in SQAT*** | | |  |
| 2 | 0.042 | GO:0004332 | fructose-bisphosphate aldolase activity |
| 10 | 0.04 | GO:0051287 | NAD or NADH binding |
| 17 | 0.002 | GO:0016616 | oxidoreductase activity, acting on the CH-OH group of donors, NAD or NADP as acceptor |
| 17 | 0.005 | GO:0016614 | oxidoreductase activity, acting on CH-OH group of donors |
| 21 | 0.002 | GO:0051186 | cofactor metabolic process |
| 20 | 0.008 | GO:0006732 | coenzyme metabolic process |
| 25 | 0.002 | GO:0050662 | coenzyme binding |
| 30 | 0 | GO:0032787 | monocarboxylic acid metabolic process |
| 33 | 0 | GO:0048037 | cofactor binding |
| 21 | 0.016 | GO:0006631 | fatty acid metabolic process |
| 27 | 0.005 | GO:0008610 | lipid biosynthetic process |
| 42 | 0 | GO:0019752 | carboxylic acid metabolic process |
| 42 | 0 | GO:0006082 | organic acid metabolic process |
| 49 | 0.002 | GO:0016491 | oxidoreductase activity |
| 44 | 0.009 | GO:0055114 | oxidation reduction |
| 45 | 0.008 | GO:0044255 | cellular lipid metabolic process |
| 52 | 0.008 | GO:0006629 | lipid metabolic process |
| 85 | 0 | GO:0005739 | mitochondrion |
| 80 | 0.002 | GO:0044248 | cellular catabolic process |
| 85 | 0.004 | GO:0009056 | catabolic process |
| 230 | 0 | GO:0044444 | cytoplasmic part |
| ***Upregulated in PCAT*** | | |  |
| 5 | 0.026 | GO:0042692 | muscle cell differentiation |
| 11 | 0.031 | GO:0009897 | external side of plasma membrane |
| 11 | 0.047 | GO:0046649 | lymphocyte activation |
| 15 | 0.005 | GO:0050865 | regulation of cell activation |
| 14 | 0.009 | GO:0006935 | chemotaxis |
| 14 | 0.009 | GO:0042330 | taxis |
| 13 | 0.048 | GO:0001775 | cell activation |
| 27 | 0 | GO:0048583 | regulation of response to stimulus |
| 24 | 0.005 | GO:0008284 | positive regulation of cell proliferation |
| 39 | 0 | GO:0002376 | immune system process |
| 26 | 0.005 | GO:0006955 | immune response |
| 21 | 0.049 | GO:0002682 | regulation of immune system process |
| 24 | 0.011 | GO:0009605 | response to external stimulus |
| 27 | 0.014 | GO:0005576 | extracellular region |
| 38 | 0 | GO:0042127 | regulation of cell proliferation |
| 26 | 0.032 | GO:0051239 | regulation of multicellular organismal process |
| 32 | 0.017 | GO:0035466 | regulation of signaling pathway |
| 62 | 0.002 | GO:0048522 | positive regulation of cellular process |
| 67 | 0 | GO:0048518 | positive regulation of biological process |
| 44 | 0.046 | GO:0042221 | response to chemical stimulus |
