# Supplementary material for: Pattern Specification and Immune Response Transcriptional Signatures of Pericardial and Subcutaneous Adipose Tissue
Source: PLoS One. 2011 Oct 11;6(10):e26092. doi: 10.1371/journal.pone.0026092 (PMC3191160; doi:10.1371/journal.pone.0026092)
Supplement: Table S2 — Upregulated Homeobox Genes in SQAT and PCAT. (DOCX) [file pone.0026092.s004.docx]

| ***Overexpressed in subcutaneous adipose tissue*** | | | | |
| --- | --- | --- | --- | --- |
|  | Gene | p-value | q-value | Fold Change |
|  | PAX3 | 0.00038 | 0.0524 | 3.84 |
|  | IRX5 | 0.00102 | 0.4747 | 1.76 |
|  | EMX2 | 0.39345 | 0.8208 | 0.36 |
|  | HOXC9 | 0.00538 | 0.1130 | 2.81 |
|  | HOXA10 | 0.00006 | 0.0253 | 3.81 |
|  | IRX3 | 0.01994 | 0.1720 | 2.25 |
|  | IRX1 | 0.00604 | 0.1161 | 2.09 |
|  | HOXB7 | 0.00226 | 0.0900 | 2.24 |
|  | HOXA9 | 0.00009 | 0.0290 | 2.96 |
|  | HOXC6 | 0.00226 | 0.0900 | 2.43 |
|  | SHOX2 | 0.00032 | 0.0463 | 2.75 |
|  | HOXC10 | 0.01330 | 0.1482 | 2.15 |
| ***Overexpressed in pericardial adipose tissue*** | | | | |
|  | Gene | p-value | q-value | Fold Change |
|  | HOXA2 | 0.00149 | 0.0772 | 2.84 |
|  | SATB1 | 0.00497 | 0.1100 | 2.03 |
