# Supplementary material for: Pattern Specification and Immune Response Transcriptional Signatures of Pericardial and Subcutaneous Adipose Tissue
Source: PLoS One. 2011 Oct 11;6(10):e26092. doi: 10.1371/journal.pone.0026092 (PMC3191160; doi:10.1371/journal.pone.0026092)
Supplement: Table S3 — Upregulated Immune Response genes in pericardial adipocytes. (DOCX) [file pone.0026092.s005.docx]

| Gene Symbol | Gene Description | p-value | q-value | Fold Change over sqAds |
| --- | --- | --- | --- | --- |
| CCL4 | Chemokine (C-C motif) ligand 4 | 0.0000 | 0.034 | 2.79 |
| BLNK | B-cell linker | 0.0005 | 0.083 | 2.27 |
| CD28 | CD28 molecule | 0.0007 | 0.097 | 2.26 |
| IGL@ | Immunoglobulin lambda locus | 0.0040 | 0.174 | 2.17 |
| PRG4 | Proteoglycan 4 | 0.0061 | 0.197 | 2.16 |
| CD274 | CD274 molecule | 0.0067 | 0.203 | 2.13 |
| LCP2 | Lymphocyte cytosolic protein 2 | 0.0008 | 0.099 | 2.07 |
| NCF1C | Neutrophil cytosolic factor 1C pseudogene | 0.0015 | 0.129 | 2.04 |
| CXCL1 | Chemokine (C-X-C motif) ligand 1 | 0.0007 | 0.099 | 2.03 |
| CST7 | Cystatin F (leukocystatin) | 0.0073 | 0.206 | 2.02 |
| CCL5 | Chemokine (C-C motif) ligand 5 | 0.0045 | 0.183 | 2.02 |
| SH2D1A | SH2 domain containing 1A | 0.0047 | 0.185 | 2.01 |
| CD8A | CD8a molecule | 0.0034 | 0.167 | 2.00 |
| CD27 | CD27 molecule | 0.0151 | 0.250 | 1.89 |
| SEMA4D | Sema domain, immunoglobulin domain, transmembrane domain and short cytoplasmic domain, 4D | 0.0055 | 0.195 | 1.88 |
| CD83 | CD83 molecule | 0.0054 | 0.194 | 1.88 |
| TNFSF9 | Tumor necrosis factor superfamily, member 9 | 0.0147 | 0.249 | 1.84 |
| POU2AF1 | POU class 2 associating factor 1 | 0.0131 | 0.243 | 1.82 |
| LY9 | Lymphocyte antigen 9 | 0.0141 | 0.247 | 1.81 |
| NCF4 | Neutrophil cytosolic factor 4, 40kDa | 0.0082 | 0.215 | 1.80 |
| CXCL5 | Chemokine (C-X-C motif) ligand 5 | 0.0136 | 0.245 | 1.77 |
| VAV1 | Vav 1 guanine nucleotide exchange factor | 0.0114 | 0.237 | 1.77 |
| CYBB | Cytochrome b-245, beta polypeptide | 0.0071 | 0.205 | 1.72 |
| IL1B | Interleukin 1, beta | 0.0029 | 0.163 | 1.70 |
| C7 | Complement component 7 | 0.0072 | 0.205 | 1.70 |
| IGKC | Immunoglobulin kappa constant | 0.0069 | 0.205 | 1.66 |
