# Supplementary material for: Pattern Specification and Immune Response Transcriptional Signatures of Pericardial and Subcutaneous Adipose Tissue
Source: PLoS One. 2011 Oct 11;6(10):e26092. doi: 10.1371/journal.pone.0026092 (PMC3191160; doi:10.1371/journal.pone.0026092)
Supplement: Table S4 — Expression of core adipocyte function genes in isolated pericardial adipocytes (pcAds) vs. isolated subcutaneous adipocytes (sqAds). (DOCX) [file pone.0026092.s006.docx]

| \| Gene \| Description \| p-value \| q-value \| Fold Change (pcAds / sqAds) \| \| --- \| --- \| --- \| --- \| --- \| \| PLIN1 \| Perilipin 1 \| 0.88 \| 0.98 \| 1.03 \| \| CIDEC \| Cell death-inducing DFFA-like effector c \| 0.03 \| 0.58 \| 0.63 \| \| PPARG \| Peroxisome proliferator-activated receptor gamma \| 0.08 \| 0.64 \| 1.49 \| \| CEBPA \| CCAAT/enhancer binding protein, alpha \| 0.98 \| 1.00 \| 1.01 \| \| CEBPB \| CCAAT/enhancer binding protein, beta \| 0.22 \| 0.75 \| 0.75 \| \| CEBPD \| CCAAT/enhancer binding protein, delta \| 0.58 \| 0.90 \| 0.88 \| \| LPL \| Lipoprotein lipase \| 0.37 \| 0.82 \| 1.27 \| \| LIPE \| Hormone-sensitive lipase \| 0.02 \| 0.57 \| 1.74 \| \| FABP4 \| Fatty acid binding protein 4 \| 0.01 \| 0.55 \| 1.91 \| \| DGAT1 \| Diacylglycerol O-acyltransferase 1 \| 0.92 \| 0.98 \| 1.03 \| \| LEP \| Leptin \| 0.01 \| 0.55 \| 1.78 \| \| CFD \| Adipsin \| 0.14 \| 0.69 \| 1.39 \| \| ADIPOQ \| Adiponectin \| 0.79 \| 0.96 \| 1.06 \| \| UCP1 \| Uncoupling protein 1 \| 0.18 \| 0.72 \| -0.50 \| |  |  |  |  |
| --- | --- | --- | --- | --- | --- | --- | --- | --- | --- | --- | --- | --- | --- | --- | --- | --- | --- | --- | --- | --- | --- | --- | --- | --- | --- | --- | --- | --- | --- | --- | --- | --- | --- | --- | --- | --- | --- | --- | --- | --- | --- | --- | --- | --- | --- | --- | --- | --- | --- | --- | --- | --- | --- | --- | --- | --- | --- | --- | --- | --- | --- | --- | --- | --- | --- | --- | --- | --- | --- | --- | --- | --- | --- | --- | --- | --- | --- | --- | --- |
